# Supplementary material for: Effects of ciprofol infusion on hemodynamics during induction and maintenance of anesthesia and on postoperative recovery in patients undergoing thoracoscopic lobectomy: Study protocol for a randomized, controlled trial
Source: PLoS One. 2024 Jul 10;19(7):e0305478. doi: 10.1371/journal.pone.0305478 (PMC11236111; doi:10.1371/journal.pone.0305478)
Supplement: S1 File — (DOCX) [file pone.0305478.s002.docx]

**Ethics Committee of The First Affiliated Hospital of Shandong First Medical University& Shandong Provincial Qianfoshan Hospital Approval report**

No: YXLL-KY-2023(042)

| Applicant/Developer Unit: **The First Affiliated Hospital of Shandong First Medical University& Shandong Provincial Qianfoshan Hospital** | | | |
| --- | --- | --- | --- |
| Clinical Research Protocol No: V5.0 | | | Drug Registration Approval No: |
| Clinical Research Leader: Xiumei Song | | | |
| Clinical Research Title: Effects of ciprofol infusion on hemodynamics during induction and maintenance of anaesthesia and on postoperative recovery in patients undergoing thoracoscopic lobectomy: study protocol for a randomized, controlled trial. | | | |
| Clinical Research Unit and Address: **The First Affiliated Hospital of Shandong First Medical University& Shandong Provincial Qianfoshan Hospital**  No. 16766, Jingshi Road, Jinan City, Shandong Province, China. | | | |
| Ethics Committee Name and Address: **Ethics Committee of** **The First Affiliated Hospital of Shandong First Medical University& Shandong Provincial Qianfoshan Hospital**  No. 16766, Jingshi Road, Jinan City, Shandong Province, China. | | | |
| Submitted material | Drug Registration Approval: Yes/No  **Clinical Research Protocol: Yes**  Researcher's Manual: Yes/No  **Informed Consent Document for Patients: Yes**  Quality inspection report for drugs: Yes/No  **Case Report Form: Yes**  **Attachments: Yes** | Review content | Applicant Qualification Certificate: **Complies with requirements**  Method of obtaining informed consent: **Appropriate**  Opinion on research protocol: **Accepted**  Routes of Review: **Routine review** |
| Summary of Ethics Committee voting results | | | |

| present members | The number of people who should attend is 15 | The actual number of people who attended is 8 | The number of people who abstained is 0 |
| --- | --- | --- | --- |

| Voting results: | Agree: 0 | Disagree: 0 | **Make necessary modifications and agree: 8** | Terminate or suspend the trial: 0 | | Dismissal: 0 |
| --- | --- | --- | --- | --- | --- | --- |
| Conclusion: | | Make necessary modifications and agree | | | | |
| The Ethics Committee approves the comments： The Ethics Committee meet on March 29, 2023 to review and discuss the project in strict accordance with the GCP principles and relevant Chinese regulations and guidelines.  **According to the voting results, the committee agreed to carry out the study of this project“Effects of ciprofol infusion on hemodynamics during induction and maintenance of anaesthesia and on postoperative recovery in patients undergoing thoracoscopic lobectomy: study protocol for a randomized, controlled trial.” from the date of approval,** the vestigators and sponsors must strictly comply with the national CFDA 《Good Clinical Practice (GCP), 》consciously accept the constraints of relevant national laws and regulations, pay attention to prevent adverse effects, and protect the rights and safety of patents. All data should not be modifed without the approval of the Committee; lf any serious adverse events occur in the study, please notify the Committee immediately. | | | | | | |
| Signature of Ethical Committee Chairman/Deputy Chairman: **Prof Dr.Tao Xin** | | | | | Date: April 10, 2023 | |
| Seal of Ethical Committee: Ethics Committee of The First Affiliated Hospital of Shandong First Medical University | | | | | | |
