# Supplementary material for: Effects of ciprofol infusion on hemodynamics during induction and maintenance of anesthesia and on postoperative recovery in patients undergoing thoracoscopic lobectomy: Study protocol for a randomized, controlled trial
Source: PLoS One. 2024 Jul 10;19(7):e0305478. doi: 10.1371/journal.pone.0305478 (PMC11236111; doi:10.1371/journal.pone.0305478)
Supplement: S2 File — (DOCX) [file pone.0305478.s003.docx]

Effect of Ciprofol Infusion for Induction and Maintenance on Hemodynamics and Postoperative Recovery in Patients Undergoing Thoracoscopic Lobectomy : Study protocol for a Randomized, Controlled Trial

**Background**

Ciprofol is a new drug candidate for induction and maintenance of anesthesia in clinical practice[1; 2]. Like propofol, the most commonly used intravenous anesthetic, ciprofol is also a receptor enhancer for gamaminobutyric acid (GABA). Several clinical trials on ciprofol has completed in China and Australia, and it has shown good efficacy and safety. Ciprofol has been widely used for aneshesia in gastroenteroscopy, fiberbronchoscopy, hysteroscopy and elective surgical induction, and has shown advantages on such as low incidence of injection pain，mild respiratory depression and stable circulation. Multiple preclinical and clinical studies have indicated that ciprofol exhibits dose-related sedative-hypnotic effects, rapid onset, rapid offset, a potency 4–6 times greater than propofol, and minor residual side effects following the administration of a single therapeutic dose[3].

At present, ciprofol can be used for endoscopy of digestive tract and induction of general anesthesia in adult patients, with a load of 0.4mg/kg, after which additional dose can be adjusted according to operation and combined anesthetic medication [4]. Yun-ying Ding’s Preliminary study have shown that general anaesthesia was successfully induced using ciprofol 0.2 to 0.4mg/kg in elderly patients undergoing major noncardiac surgery,without any serious adverse events[5] . The study by Ben zhen Chen et al. found that in gynecological surgery, ciprofol is as effective as propofol when it is used for induction of general anesthesia, while the incidence of adverse events is lower[3] . Qin K[6]et al. found that ciprofol can be used safely and effectively for the induction and maintenance in patients undergoing kidney transplant, and has a better sedative effect than propofol.

Due to the short time clinical application of ciprofol, there are few clinical studies on its use for maintenance of general anesthesia. Liang P et al. conducted a multicenter study on efficacy and safety of ciprofol vs propofol for the induction and maintenance of general anaesthesia, found that ciprofol had good efficacy and showed excellent tolerance compared with propofol[7]. However, the selected subjects were non-cardiothoracic and non-brain patients undergoing elective surgery. It is well known that thoracoscopic lobectomy often requires double-lumen endotracheal intubation, with strong stimulation response and high intraoperative risk. Induction and maintenance of patients with thoracoscopic lobectomy require appropriate depth of anesthesia. Whether ciprofol can be successfully used for induction and maintenance of patients with thoracoscopic lobectomy remains unclear. What is the effect of ciprofol on such patients still unclear. Propofol is one of the commonly used drugs for induction and maintenance of general anesthesia, which is favored by anesthesiologists for its advantages of good recovery, short half-life, rapid elimination from blood circulation, small residual sedation and low incidence of vomiting [8]. However, its most serious side effects are hemodynamic instability and cardiovascular complications, such as hypotension [9]. As we all know, maintaining stable hemodynamics during anesthesia is crucial to the prognosis of patients. As a new type of intravenous anesthesia drug, can ciprofol maintain more stable hemodynamics compared with propofol? In this project, it is the first time to study the effects of ciprofol on hemodynamics and postoperative recovery in patients undergoing thoracoscopic lobectomy.

**Methods/design**

**Study design**

Approved by the Ethics Committee of the First Affiliated Hospital of Shandong First Medical University's Ethics Committee (YXLL-KY-2023(042)), the randomized, controlled trial has been registered with the clinicaltrials.gov (NCT05664386). It is done in accordance with the guidelines of good clinical practice and the Declaration of Helsinki[10; 11]. It complies with Standard Protocol Items: Recommendations for Interventional Trials (SPIRIT)[12].

The trial will be performed at the First Affiliated Hospital of Shandong First Medical University, located in Jinan City, Shandong Province. A total of 126 participants will be assigned to either propofol group (n=63) or ciprofol group (n=63) randomly. The actual start date of the study is April 2023 and the expected completion date is April 2024.The study design is shown in Figure 1.

Assessed for eligibility (n= )

Excluded (n= )

♦  Predictable airway difficulties (n= )

♦  Declined to participate (n= )

♦  Patients with mental diseases or consciousness disorder (n= )

♦  Tracheal intubation failed for twice (n= )

♦  Long-term use of sedatives or Antidepressants(n=)

Analysed (n= )
♦ Excluded from analysis (give reasons) (n= )

## Analysis

## Follow-Up

## Enrollment

Systolic blood pressure (SBP) , diastolic blood pressure (DBP), mean arterial pressure (MAP) and heart rate (HR) during induction and maintenance(T_0_-T_6_,OR_0_-OR_5_OR_6_……)

Propofol Group (n= )

Ciprofol Group (n= )

Allocated to Ciprofol Group (n= )

♦ Received allocated Ciprofol Group (n= )

♦ Did not receive allocated Ciprofol Group (give reasons) (n= )

Allocated to Propofol Group (n= )

♦ Received allocated Propofol Group (n= )

♦ Did not receive allocated Propofol Group (give reasons) (n= )

## Allocation

Systolic blood pressure (SBP) , diastolic blood pressure (DBP), mean arterial pressure (MAP) and heart rate (HR) during induction and maintenance(T_0_-T_6_,OR_0_-OR_5_OR_6_……)

Lost to follow-up (give reasons) (n= )

Discontinued intervention (give reasons) (n= )

## Evaluate

Lost to follow-up (give reasons) (n= )

Discontinued intervention (give reasons) (n= )

FIGURE 1

General review of study design (flow diagram)

Analysed (n= )
♦ Excluded from analysis (give reasons) (n= )

**Sample size estimation**

The primary outcome of this study is perioperative hemodynamic fluctuation, and the fluctuation of mean arterial pressure(MAP) was used as evaluation index. According to the results of the preliminary experiment, the standard deviation of each group was about 9, and the autocorrelation coefficient between adjacent measurement points of the same study participant was 0.3. In the pre-test, the difference of MAP between the two groups was 2.48, α=0.05, 1-β=80%, bilateral test, the sample size of the two groups was equal, and the sample size of each group was 50. Considering the 20% shedding rate, the sample size was 63 cases in each group, and the final sample size was 126.

**Eligibility criteria**

Inclusion criteria:

1. American Society of Anesthesiology (ASA): Grade Ⅰ~Ⅱ;
2. aged 18-65 years;
3. body mass index (BMI) of 20-30kg/m^2^ ;
4. scheduled for thoracoscopic lobectomy under general anesthesia.

Exclusion criteria:

1. Patients and their families refused to participate in the clinical study;
2. Predictable airway difficulties requiring awake intubation;
3. Allergy to the drugs which were used in this study;
4. tracheal intubation failed for twice;
5. Patients with mental diseases or consciousness disorder;
6. long-term use of sedatives or Antidepressants;
7. Patients who are participating in other clinical studies.

**Shedding criteria**

1. Severe intraoperative massive bleeding (more than 2000ml of blood loss);
2. Patients with serious cardiovascular events (such as malignant arrhythmia, acute myocardial ischemia);
3. Patients with anaphylactic shock;
4. The thoracoscopic surgery become thoracotomy.
5. **The patient or the client refuses the informed consent or requests to withdraw from the study during the observation period.**

**Study implementation**

**Anesthesia and monitoring methods:** All participants will be fasted for 8 h before surgery, and water is forbidden for 2h. After entering the operation room, an intravenous catheter will be placed in the forearm of the subjects for drug administration and intravenous infusion of compound sodium chloride injection. The participants will be continuously monitored using ECG, pulse oxygen saturation, end-tidal carbon dioxide concentration, non-invasive blood pressure, and the bispectral index (BIS) of electroencephalography (EEG). Radial artery catheterization will be performed to monitor invasive blood pressure. Other monitoring items (central venous pressure, cerebral oxygen saturation, etc.) will be added according to the actual situation. A 35F or 37F left double-lumen tracheal catheter will be selected according to height and sex. According to the random number , the participants will be divided into group （propofol group）and group C（ciprofol group）. The two protocols are detailed in Table 1.

**Intravenous anesthesia induction:** The induction period of general anaesthesia is deﬁned as the time from the injection of ciprofol or propofol to 5 minutes after the completion of tracheal intubation. During the induction process, compound sodium chloride injection will be continuously injected at a rate of 8 ~ 10ml/kg/h while the subjects are breathing 6 L/min of oxygen via a mask. All patients will not receive midazolam.The group P will receive propofol 2～2.5mg/kg for anesthesia induction , the group C will receive ciprofol 0.4 ~ 0.5mg/kg for induction. If BIS value ≤60, cisatracurium 0.2mg/kg and sufentanil 0.5μg/kg will be injected, and double-lumen endotracheal intubation will be performed after muscle relaxed. If BIS value >60, propofol (1mg/kg) or ciprofol (0.2mg/kg) will be added intravenously, and the interval of administration is >1min, until BIS≤60. Cisatracurium 0.2mg/kg and sufentanil 0.5μg/kg will be injected, and double-lumen endotracheal intubation will be performed after muscle relaxed. When the endotracheal intubation is completed, both lungs will be ausculted to check the position of the double-lumen tubes preliminarily and determine whether bronchospasm has occurred. If bronchospasm occurs, the depth of anesthesia will be increased. Next, the anesthetist will fix the tracheal intubation tube and maintenance drug infusion will be started 5 minutes after the completion of tracheal intubation. Then the patient will be placed in lateral decubitus position, fibrobronchoscope will be used for localization. Volume-controlled ventilation (respiratory rate is set at 10 ~ 14 times /min, tidal volume at 6 ~ 8ml/kg, suction/breathing ratio at 1:2, oxygen flow rate at 2L/min) will be performed after intubation, and PaCO_2_ is maintained at 35 ~ 45 mmHg. When systolic blood pressure decreases by more than 30% or less than 90 mmHg during induction, the infusion speed will be accelerated and vasoactive drugs will be given. If the heart rate is lower than 40 beats /min or higher than 100 beats /min, atropine or esmolol will be given symptomatic treatment.

**Anesthesia maintenance**: The maintenance is deﬁned as the time from initiation to the discontinuation of the experimental drugs. The maintenance drugs will be started 5 minutes after the completion of tracheal intubation. Propofol will be initially given at 4.0 mg/kg/h and ciprofol will be initially given at 0.8 mg/kg/h respectively. The group P will receive continuous propofol infusions at a rate of 4 ~ 12mg/kg/h for maintenance. The group C will receive continuous ciprofol infusions at a rate of 0.8 ~ 2.5mg /kg/h for maintenance. Remifentanil will be administered at 0.1–0.3ug/kg/min for analgesia in both groups. At the beginning of the operation, single lung ventilation will be started and respiratory parameters will be adjusted to maintain PaCO_2_ of 35 ~ 45 mmHg. Cisatracurium and sufentanil will be added according to the duration and stimulation of the surgery. The total amount dosage of sufentanil will be controlled at 0.7ug ~ 1ug/kg. Intraoperative BIS will be maintained at 40 ~ 60, blood pressure will be controlled by vasoactive drugs when necessary. Experimental drug administration will be stopped when surgical procedures are completed.

**During the PostAnesthesia Care Unit(PACU):** All patients will be transferred to the post-anesthesia care unit after the surgery. Ramsay score will be recorded when patients are transferred to PACU after surgery. The time to recovery of consciousness, time to recovery of spontaneous respiration and extubation time will be recorded. Ricker Sedation-Agitation Scale (SAS)and Ramsay score at extubation will be recorded. Numeric Rating Scale (NRS) score after recovery will be recorded. If NRS score > 3, Sufentanil 0.1ug/kg will be given. During the PACU period, postoperative nausea and vomiting(PONV) and the use of remedial drugs will be recorded. Patients who meet the PACU exit indication will be transferred to the ward, PACU residence time will be recorded . Patients will be followed up on the first and second day after surgery, NRS scores and PONV will be recorded . Quality of Recovery score (QOR)-15 questionnaire[13] will be used to assess quality of recovery before surgery, on the first and second day after surgery. Brice questionair will be used to assess intraoperative awareness on the first day after surgery.( supplementary materials)

**Exposures**

The group P will receive propofol 2～2.5mg/kg for anesthesia induction ,the group C will receive ciprofol 0.4 ~ 0.5mg/kg for induction.

The group P will receive continuous propofol infusions at a rate of 4 ~ 12mg/kg/h for maintenance. The group C will receive continuous ciprofol infusions at a rate of 0.8 ~ 2.5mg /kg/h for maintenance. Remifentanil will be administered at 0.1–0.3ug/kg/min for analgesia in both groups. Sufentanil and cisatracurium will be added according to the duration and stimulation of the surgery.

**Outcome**

**Primary outcomes**

Hemodynamic changes during anesthesia induction and maintenance:

Systolic blood pressure (SBP) , diastolic blood pressure (DBP), mean arterial pressure (MAP) and heart rate (HR) will be recorded before induction (T0), immediately before endotracheal intubation (T1), 1min after endotracheal catheter insertion (T2), 3min after endotracheal intubation (T3), 5min after endotracheal intubation (T4),when the patient is placed in the lateral decubitus position (T5), When the anesthesiologist  using fiberoptic bronchoscope for localization(T6). SBP, DBP, MAP and HR will be recorded right before the surgery(OR0),1 min after the operation begin（OR1）, 3 min after the operation begin（OR2）, 5 min after the operation begin（OR3）, 30min after the operation begin(OR4), then every 30 min (OR5,OR6………….) until the end of the operation.

**The secondary outcomes**

(1) The time to loss of consciousness (T_LOC_), the times and dosage of additional drug, and the change of BIS during induction.

(2) The incidence of injection pain, body movement, muscle twitching, hypotension, hypertension, bradycardia，tachycardia, cough and bronchospasm. Hemodynamically unstable treatments during induction and maintenance.

(3) Quality of Recovery score（QOR15）will be recorded on the day before surgery, postoperative day 1 (POD1) and postoperative day 2 (POD2).

(4) The time to recovery of consciousness (T_ROC_), time of respiration recovery and extubation time will be recorded. PACU residence time, length of hospital stay (LOS)after surgery .

(5) Incidence and severity of postoperative nausea and vomiting, postoperative agitation and delayed recovery, and intraoperative awareness.

The time to loss of consciousness (T_LOC_) is defined as time from the first drug administration(ciprofol or propofol) to BIS＜60. The time to recovery of consciousness (T_ROC_) is defined as from the discontinuation of the experimental drugs to response to verbal commands and sufficient spontaneous breathing.

QoR-15 questionnaire, which is a global measure of recovery after surgery that evaluates five dimensions of recovery: physical comfort (5items),physical independence (2 items), emotional state (4 items), psychological support (2 items), and pain (2 items). Each item is rated on an 11point scale based on its frequency on the questionnaire (greater score at greater frequency for positive items and less frequency for negative items). The total score ranged from 0 (poorest recovery quality) to 150 (best recovery quality). The patients will complete the QoR-15 questionnaire at three time points: the day before surgery; POD1; and POD2[14]. Study timeline and schedule of enrolment, allocation, interventions, and assessments according to SPIRIT 2013 statement (showed in table 2).

**Informed consent**

The researchers obtained consent from the patients before surgery. The investigator is responsible for explaining to the study participants the purpose, methods, expected benefits, and potential risks of the this clinical trial, and obtaining a signed informed consent form from each participant. If the participants are unable to sign a legal informed consent, they must require their legal representatives to sign a written informed consent. The investigator or designee must explain to the participants that they have the right to decline to participate in the study and to withdraw from the study at any time for any reason. If new safety studies indicate significant changes in the benefit-risk assessment, the informed consent should be reviewed and, if necessary, modified. All study participants must know the new study information. Participants will receive an amended informed consent form. The researchers should obtain their informed consent to continue the study. Study participants will be screened based on inclusion/exclusion criteria after signing an informed consent form (ICF).

**Sampling**

(1) Patients are numbered as 1, 2, 3...... 126, according to the order of enrollment.

(2) Obtain random numbers: Start from any number in the random number table, and obtain a random number for each patient according to entry sequence in the same direction.

(3) Remainder: the remainder of the random number divided by the number of groups can be evenly divided into the propofol group, but cannot be evenly divided into the ciprofol group.

(4) Adjustment: if the number of cases in the two groups is not equal, the extra patients in one group can continue to check the random number table and find the surplus again Count until the two groups are equal. Randomization is performed by a permanent nurse anesthesiologist.

(5) The random number generated is placed in an opaque sealed bag by the fixed nurse anesthesiologist and handed over to the fixed nurse anesthesiologist in the drug preparation room, who prepares the corresponding drug according to the group.

**Data management**

1) The test system and the equipment for clinical research should be verified regularly;

2) All researchers should operate follow SOP and study protocol rigorously;

3) Recording data should be timely, direct, accurate and clear, signed and dated;

4) Always check the accuracy and completeness of data records, and correct errors in accordance with the prescribed methods;

5) Patients and personnel training shall be included in strict accordance with inclusion and exclusion criteria;

6) Verified and reliable statistical software is adopted for statistical processing of data, and effective quality control measures are adopted for data input, such as double or double entry (lose-lose method, double entry), etc.

**Statistical analysis**

1. The measurement data conforming to the normal distribution are expressed by mean ± standard deviation(x ± s). Methods Repetitive measure analysis of variance (ANOVA) will be used in analyzing the repeated measurement data (blood pressure , heart rate, NRS score) compared within the group. The independent t-test or one-way ANOVA are used for inter-group comparison.
2. The measurement data of non-normal distribution are expressed by median (m) and 25th and 75th percentile (P25, p75). Mann-Whitney U test is used for comparison between groups.
3. Categorical variables(such as injection pain, muscle twitching, cough, bronchospasm, postoperative agitation, nausea and vomiting, delayed recovery, and intraoperative awareness）will be described as counts(percentages) and compared using χ2 analysis or Fisher’s exact test.
4. The independent t-test or the Wilcoxon rank sum test will be used for the comparison of numerical variables（such as time to loss of consciousness, time to recovery of consciousness, time of extubation, time of PACU residence and length of hospital stay） between two groups.
5. The overall significance level is set at p < 0.05 and Bonferroni correction will be used to control type I errors.

**Reference**

[1] L. Qin, L. Ren, S. Wan, G. Liu, X. Luo, Z. Liu, F. Li, Y. Yu, J. Liu, and Y. Wei, Design, Synthesis, and Evaluation of Novel 2,6-Disubstituted Phenol Derivatives as General Anesthetics. J Med Chem 60 (2017) 3606-3617.

[2] Y. Bian, H. Zhang, S. Ma, Y. Jiao, P. Yan, X. Liu, S. Ma, Y. Xiong, Z. Gu, Z. Yu, C. Huang, and L. Miao, Mass balance, pharmacokinetics and pharmacodynamics of intravenous HSK3486, a novel anaesthetic, administered to healthy subjects. Br J Clin Pharmacol 87 (2021) 93-105.

[3] B.Z. Chen, X.Y. Yin, L.H. Jiang, J.H. Liu, Y.Y. Shi, and B.Y. Yuan, The efficacy and safety of ciprofol use for the induction of general anesthesia in patients undergoing gynecological surgery: a prospective randomized controlled study. BMC Anesthesiol 22 (2022) 245.

[4] Y. Teng, M. Ou, X. Wang, W. Zhang, X. Liu, Y. Liang, K. Li, Y. Wang, W. Ouyang, H. Weng, J. Li, S. Yao, J. Meng, W. Shangguan, Y. Zuo, T. Zhu, B. Liu, and J. Liu, Efficacy and safety of ciprofol for the sedation/anesthesia in patients undergoing colonoscopy: Phase IIa and IIb multi-center clinical trials. Eur J Pharm Sci 164 (2021) 105904.

[5] Y.Y. Ding, Y.Q. Long, H.T. Yang, K. Zhuang, F.H. Ji, and K. Peng, Efficacy and safety of ciprofol for general anaesthesia induction in elderly patients undergoing major noncardiac surgery: A randomised controlled pilot trial. Eur J Anaesthesiol 39 (2022) 960-963.

[6] K. Qin, W.Y. Qin, S.P. Ming, X.F. Ma, and X.K. Du, Effect of ciprofol on induction and maintenance of general anesthesia in patients undergoing kidney transplantation. Eur Rev Med Pharmacol Sci 26 (2022) 5063-5071.

[7] P. Liang, M. Dai, X. Wang, D. Wang, M. Yang, X. Lin, X. Zou, K. Jiang, Y. Li, L. Wang, W. Shangguan, J. Ren, and H. He, Efficacy and safety of HSK3486 vs. propofol for the induction and maintenance of general anaesthesia: A multicentre, single-blind, randomised, parallel-group, phase 3 clinical trial. Eur J Anaesthesiol (2023).

[8] U. Kondo, S.O. Kim, and P.A. Murray, Propofol selectively attenuates endothelium-dependent pulmonary vasodilation in chronically instrumented dogs. Anesthesiology 93 (2000) 437-46.

[9] M. Masoudifar, and E. Beheshtian, Comparison of cardiovascular response to laryngoscopy and tracheal intubation after induction of anesthesia by Propofol and Etomidate. J Res Med Sci 18 (2013) 870-4.

[10] J.E. Idänpään-Heikkilä, WHO guidelines for good clinical practice (GCP) for trials on pharmaceutical products: responsibilities of the investigator. Ann Med 26 (1994) 89-94.

[11] World Medical Association Declaration of Helsinki: ethical principles for medical research involving human subjects. J Am Coll Dent 81 (2014) 14-8.

[12] A.W. Chan, J.M. Tetzlaff, P.C. Gøtzsche, D.G. Altman, H. Mann, J.A. Berlin, K. Dickersin, A. Hróbjartsson, K.F. Schulz, W.R. Parulekar, K. Krleza-Jeric, A. Laupacis, and D. Moher, SPIRIT 2013 explanation and elaboration: guidance for protocols of clinical trials. Bmj 346 (2013) e7586.

[13] J. Kleif, J. Waage, K.B. Christensen, and I. Gögenur, Systematic review of the QoR-15 score, a patient- reported outcome measure measuring quality of recovery after surgery and anaesthesia. Br J Anaesth 120 (2018) 28-36.

[14] J.Y. Choi, H.S. Lee, J.Y. Kim, D.W. Han, J.Y. Yang, M.J. Kim, and Y. Song, Comparison of remimazolam-based and propofol-based total intravenous anesthesia on postoperative quality of recovery: A randomized non-inferiority trial. J Clin Anesth 82 (2022) 110955.
