# Supplementary material for: Effects of ciprofol infusion on hemodynamics during induction and maintenance of anesthesia and on postoperative recovery in patients undergoing thoracoscopic lobectomy: Study protocol for a randomized, controlled trial
Source: PLoS One. 2024 Jul 10;19(7):e0305478. doi: 10.1371/journal.pone.0305478 (PMC11236111; doi:10.1371/journal.pone.0305478)
Supplement: Attachment — Submitted filename: Response to Reviewers.docx [file pone.0305478.s004.docx]

Dear Editor and Reviewers:

Thank you for your letter and for the reviewers’ comments concerning our manuscript entitled “Effects of ciprofol infusion on hemodynamics during induction and maintenance of anaesthesia and on postoperative recovery in patients undergoing thoracoscopic lobectomy: study protocol for a randomized, controlled trial”.
(Manuscript ID: PONE-D-23-32710).

Those comments are all valuable and very helpful for revising and improving our paper, as well as the important guiding significance to our researches. We have studied comments carefully and have made correction which we hope meet with approval. The reviewers’ comments are laid out below in italicized font and specific concerns have been numbered. Our response is given in normal font.

Revised portion are marked in red in the paper.

**Reviewer 1**

1. **Comment:** *Reviewer #1: This is a well-designed study, testing a new drug, currently approved for use in China.
   My suggestions for improvement:
   - If the study is a trial protocol - it should be published before the start of the study, from this protocol in certain places one gets the impression that the study has already been completed, e.g.
   - The length of stay in the PACU was at least 30 minutes
   - The authors declare that the research was conducted...
   - The patients/participants provided their written informed consent...
   It is necessary to make it uniform, please specify.*

**Response:** Thank you for your rigorous consideration. We need to explain that due to several reasons, the planned completion date of this study is delayed to December 2024. We have revised the “The study has been initiated in April 2023, and the expected completion date is April 2024.” to “The study has been initiated in April 2023 and the expected completion date is December 2024.”(Page 6 Line 104).

---We have changed this sentence “*The length of stay in the PACU was at least 30 minutes*” to “The length of stay in the PACU will be at least 30 minutes after removing endotracheal tubes.” (Page 10, Line 204 2.6..4 Recovery of anesthesia )

The revised portion are marked in red.

------We have revised this “ The authors declare that the research was conducted” to “The authors declare that the research will be conducted”, marked in red. (page 2 ,Line 23 Conflict of interest)

---*-“The patients/participants provided their written informed consent... ”*this sentence was originally written in that paragraph “Line 475 Ethics statement ”. According to format requirements of the journal, we deleted this part . We have rewritten the part “Ethical considerations and declarations” in page 17 line 333-341, 2.13 Ethical considerations and declarations. We are very sorry for our mistake. We have revised the tense.

All the revised portion are marked in red in the paper. We gratefully appreciate for your valuable suggestion.

2. ***Comment:*** *LN. 313 2.10 Sampling - replace this word with "Randomization", because Sampling usually refers to taking samples (laboratory, microbiology, etc.)*

**Response:** We gratefully appreciate for your valuable comment. We have made correction according to the Reviewer’s comments. ----page 15, line 288 2.10 we replaced “Sampling” with “Randomization and blinding”. Thank you so much for your careful check.

3. ***Comment:*** *According to the protocol for the RCT statement in Supplemental file S1:
   - 21b Description of any interim analyzes and stopping guidelines, including who will have access to these interim results and make the final decision to terminate the trial NA
   This statement is a necessary part of ethical standards. Data on the safety of ciprofol exist in other categories of patients. However, the possibility of serious side effects related to the use in a special category of patients - thoracic surgery. The authors justified the conduct of this study with a new category of patients. According to the ethical standards - each study manager must terminate the trial in the event of serious adverse events and inform the Ethics Committee accordingly. I propose to include that statement in the Ethical standards.*

**Response:** Thank you for your rigorous consideration. We have revised the text to address your concerns and hope that it is now clearer. In “2.13 Ethical considerations and declarations” paragraph, we have included the statement “The investigator must report all Serious Adverse Events(SAEs) to the sponsor (the correspoding author in this trial ) immediately and inform the Ethics Committee accordingly. The sponsor is responsible for ongoing safety evaluation of the investigational drug (ciprofol and propofol), terminating the trial in the event of SAEs and compensating the participant in case of any SAE. ” (Page 17-18, Line 334-343 )

In “ **2.11 Data management ”,** we have added this paragraph “Investigators will conduct interim analyse to make informed decisions about whether to continue the trial or revise the study design to optimize the trial outcomes.” (Page16, Line 317-318)

---We revised the Supplemental file S1 “ 21b Description of any interim analyzes and stopping guidelines, including who will have access to these interim results and make the final decision to terminate the trial. NA ” to “ 21b Description of any interim analyzes and stopping guidelines, including who will have access to these interim results and make the final decision to terminate the trial. 16-18 ”

4. ***Comment:*** *Given that the Discussion is rather unclear, I suggest that the authors structure it in such a way that they first discuss the possible gains of these studies in relation to the tested drug - ciprofol, then how the comparative drug - propofol - performed in that area, and in which segment ciprofol could be better . If the expected effect is equal to the hemodynamic effect, what would be the benefit of introducing a new drug as an alternative or standard method. Is it the price, faster elimination or something else.
   The manuscript has a number of minor grammatical errors, such as capitalizing a noun within a sentence throughout the text. Additional proofreading is therefore required.*

**Response:** Thank you so much for your careful check and nice suggestion.

------We've made extensive changes to this section in manuscript according the reviewer’s comments(First discuss the possible gains of these studies in relation to ciprofol, then how the propofol performed , next, which part the ciprofol could be better than propofol ), please see page18-20，Line 345-381. We appreciate for reviewers’ warm work earnestly, and hope that the correction will meet with approval.

----We are very sorry for incorrect writing. We have carefully corrected some grammatical errors.

Other changes: page 9, Line 182 , “The infusion of anesthetic agents will be stop immediately after the surgical procedures” were changed as “The infusion of anesthetic agents will be stopped immediately at the end of surgery.”

-----And here we did not list the changes but marked in red in revised paper.

Once again, thank you very much for your comments and suggestions.

**Reviewer 2**

1. ***Comment:*** *Reviewer #2: This protocol is for a randomised controlled trial of ciprofol vs propofol in thorascopic lobectomy. The tense of the protocol, especially the abstract, varies, and could usefully be tidied up.*

**Response:** We are very sorry for our incorrect writing.

**-**-------In Abstact part, page 2, line 33-34 “Therefore, this study aimed to observe the effects of ciprofol on hemodynamics and on postoperative recovery in patients undergoing thoracoscopic lobectomy.” were changed as “Therefore, this study aims to observe the effects of ciprofol on hemodynamics and on postoperative recovery in patients undergoing thoracoscopic lobectomy.”

Other changes were marked in red in revised paper.

2. ***Comment:*** *We are told there are few studies in this area - but none are given in the motivation - it is important here to know that the research is needed and is not merely a means to gaining more experience with ciprofol.*

**Response:** Thank you for your rigorous consideration.

We have revised abstract ,introduction and discussion section carefully. Ciprofol ,a promising intravenous anesthetic drug,is newly developed, has advantages of better tolerance, higher sedation satisfaction score, and lower incidence of adverse reactions, especially in reducing the incidence of injection pain. Some clinical trials also demonstrate that ciprofol has less effect on respiratory and circulation compared with propofol in patients undergoing noncardiacthoracic surgery, we aim to investigate the effect of ciprofol on hemodynamics and postoperative recovery.

Please refer to the revised manuscript file with tracked changes.

----We appreciate for Reviewers’ warm work earnestly, and hope that the correction will meet with approval.

3. ***Comment:*** ---- *To ethically perform a randomised study there needs to be sufficient uncertainty to allocate to either arm and this uncertainty is not expressed in the protocol.*

**Response:** We totally understand the reviewer’s concern.

------In the part of 2.10 Randomization and blinding we have written that “ The generated random allocation will be placed in an opaque sealed bag and handed over to the nurse in the drug preparation room who prepares the corresponding drug according to the group.”

------We added the contents about blinding, “The patients will be blinded to the group allocation in this study, whereas, anesthesia providers who will be unblinded to facilitate intraoperative anesthesia management. Another blinded independent researcher will be responsible for preoperative visit and obtaining informed consent with participants. The outcome assessors and the data analysts will also be blinded to the allocation .”（Page 16,line 302-306）.

***4.Comment:*** *I cannot arrive at the same size given using the data given here. For a single measurement of difference with mean 2.48 and standard deviation 9 this is a standardised difference of about 0.28 - assuming bilateral manes a two-sided test then this requires over 200 patients per group. A sample size of 50 per group is equivalent to a standardised difference of about 5/9 - this calculation needs to be made clear and the method for dealing with apparently repeated measures given here as well.*

**Response:** We feel sorry for the inconvenience brought to the reviewer. For this part of 2.2 Sample size estimation, Page 6, line 112-113, we added this description “We use a repeated measures analysis of variance (ANOVA) to calculate the sample size in the PASS 2021 (v21.0.3)”

The computational process as follows :

5. ***Comment:*** *Terminology needs to be clear - I think shedding criteria are withdrawal criteria but there is a big difference in the reasons here in terms of interpretation - how is this going to be allowed for in the ITT analysis?*

**Response:** We totally understand the reviewer’s concern. We have redefined the appropriate inclusion and exclusion criteria, withdrawal criteria. Intention-to-treat (ITT) analysis aims to include all participants randomized into a trial irrespective of what happened subsequently. We perform withdrawal criteria for patients who meet the inclusion and exclusion criteria ,but withdraw from the trial because of personal events or unpredictable events, including tracheal intubation failed twice or more，intraoperative massive bleeding, anaphylactic shock, or **request to withdraw from the study,and so on. We also added this sentence** “**These participants will no longer be included in the analysis.”** We hope that our corrections and explanations will meet with approval. (page 7,line121-140)

6. ***Comment:*** *See alos 2.10(3) for lack of clarity.*

**Response:** We feel sorry for the inconvenience brought to the reviewer. We have changed “The remainder of the random number divided by the number of groups can be evenly divided into the propofol group but cannot be evenly divided into the ciprofol group.” to “ Participants with odd numbers will be assigned to the propofol group and those with even numbers will be assigned to the ciprofol group. ”(Page 15,line294-295) We hope our revised content can be clearly expressed.

7. ***Comment:*** *Line 258 implies the list of datapoints is incomplete and the primary outcome is not defined here.*

**Response:** We are very sorry for not writing clearly. We have changed “Hemodynamic changes during anesthesia induction and maintenance.” to “The primary outcomes are mean arterial pressure (MAP) and heart rate (HR) at following time points during anesthesia induction and maintenance.” (page 12，line 233). We have added the time point---at the end of surgery(OR_10_) to ensure the completeness of datapoints.

8. ***Comment:*** *Intergroup and between group are the same thing so there are three tests here and it is not remotely clear what is being done when to which variable. The method of using Bonferroni correction is entirely unclear here - what is the multiple testing?*

**Response:** We apologize for our errors. We thank the reviewer for pointing this out.

-------We have revised as follows: “Repetitive measure analysis of variance (ANOVA) and Generalized estimating equations (GEE)will be employed to analyze the repeated measurement data, including blood pressure and heart rate.Repetitive measure analysis of variance (ANOVA) will be employed to analyze the QoR-15 scores. The measurement data that conform to a normal distribution are expressed as the mean ± standard deviation ($\bar{x}$±s).Non-normally distributed measurement data are expressed using the median and the 25^th^ and 75^th^percentiles. Numerical variables (e.g., NRS score for pain, SAS score, Ramsay score,T_LOC_, T_ROC_, extubation time, and PACU time) will be compared between the two groups using the independent t-test or Wilcoxon rank-sum test. Categorical variables (e.g., injection pain, muscle twitching, cough, postoperative agitation, nausea and vomiting, and intraoperative awareness)will be presented as counts(percentages) and compared using χ2 analysis or Fisher’s exact test. The overall significance level was set at p < 0.05.” (page 16-17, line319-332)

-----We delete the wrong description , such as “An independent t-test or one-way ANOVA will be used for intergroup comparisons. The Mann–Whitney U test will be used for comparison between the groups”. “and Bonferroni correction will be used to control for type I errors” .

We have studied comments carefully and have made correction which we hope meet with approval.

We would like to thank the reviewers again for taking the time to review our manuscript.
