# Supplementary material for: Effects of ciprofol infusion on hemodynamics during induction and maintenance of anesthesia and on postoperative recovery in patients undergoing thoracoscopic lobectomy: Study protocol for a randomized, controlled trial
Source: PLoS One. 2024 Jul 10;19(7):e0305478. doi: 10.1371/journal.pone.0305478 (PMC11236111; doi:10.1371/journal.pone.0305478)
Supplement: Attachment — Submitted filename: Response to Reviewers.docx [file pone.0305478.s005.docx]

Dear Editor and Reviewers:

Thank you for your letter and for the reviewers’ comments concerning our manuscript entitled “Effects of ciprofol infusion on hemodynamics during induction and maintenance of anaesthesia and on postoperative recovery in patients undergoing thoracoscopic lobectomy: study protocol for a randomized, controlled trial”.
(Manuscript ID: PONE-D-23-32710R1).

Those comments are all valuable and very helpful for revising and improving our manuscript, as well as the important guiding significance to our researches. We have studied comments carefully and have made correction which we hope meet with approval. The reviewers’ comments are laid out below in italicized font and specific concerns have been numbered. Our response is given in normal font.

Revised portion are marked in red in the paper.

**Reviewer 1**

**Response to reviewer 1 :** Thank you for your rigorous consideration once again.

We invited professional native English speakers to guide and carefully revise our manuscript.-----And here we did not list the changes but marked in red in revised paper. Once again, thank you very much for your comments and suggestions.

**Reviewer 2**

***1.Comment:*** *I am unclear about some of the changes here - the dataset was previously to be made available upon application - now it is to uploaded to a website (the sentence here still needs attention). Clinicaltrials.gov will accept the results, but what about the underlying individual patient data?*

**Response:** We thank the reviewer for pointing this out. We have removed the original description and replaced it with “No datasets were generated or analysed during the current study now. Data supporting this study will be available from [https://figshare.com/ at DOI:10.6084/m9.figshare.25421503, when](https://figshare.com/%20at%20DOI:10.6084/m9.figshare.25421503,%20when) the clinical trial is completed.” (Page 2,Line 27-29)
***2. Comment:*** *The English generally still needs considerable attention, and the literature search still talks about such things as &quot;fewer literatures&quot; - what does this mean? What level of evidence is there here? Have there been any studies including patients with thoracoscopic surgery? What is current standard of care here?*
**Response:** We apologize for our errors. We thank the reviewer for pointing this out. We have change “fewer literatures” to “few studies”. (Page 4,Line 82). A series of clinical studies have been conducted to evaluate the sedative effect of ciprofol in various procedures and settings, including gastroscopy and colonoscopy, fiber-optic bronchoscopy[1, 2], general anesthesia in elective surgeries, and mechanical ventilation in intensive care units[3]. Some studies of ciprofol for anesthesia induction did not clearly point out whether these studies included thoracic surgery patients [4] [5].Other studies on the induction of ciprofol for elective surgery specifically excluded thoracic surgery patient [6-11]. For all we know, although part of the studies on the induction of ciprofol included thoracic surgery patients, there were no studies of ciprofol for anesthesia maintenance in thoracic surgery.

1.	Wu B, Zhu W, Wang Q, Ren C, Wang L, Xie G. Efficacy and safety of ciprofol-remifentanil versus propofol-remifentanil during fiberoptic bronchoscopy: A prospective, randomized, double-blind, non-inferiority trial. Front Pharmacol. 2022;13:1091579. Epub 2023/01/10. doi: 10.3389/fphar.2022.1091579. PubMed PMID: 36618929; PubMed Central PMCID: PMCPMC9812563.

2.	Luo Z, Tu H, Zhang X, Wang X, Ouyang W, Wei X, et al. Efficacy and Safety of HSK3486 for Anesthesia/Sedation in Patients Undergoing Fiberoptic Bronchoscopy: A Multicenter, Double-Blind, Propofol-Controlled, Randomized, Phase 3 Study. CNS Drugs. 2022;36(3):301-13. Epub 2022/02/15. doi: 10.1007/s40263-021-00890-1. PubMed PMID: 35157236; PubMed Central PMCID: PMCPMC8927014 have no conflict of interest.

3.	Lu M, Liu J, Wu X, Zhang Z. Ciprofol: A Novel Alternative to Propofol in Clinical Intravenous Anesthesia? Biomed Res Int. 2023;2023:7443226. Epub 2023/01/31. doi: 10.1155/2023/7443226. PubMed PMID: 36714027; PubMed Central PMCID: PMCPMC9879693 personal relationships that could have appeared to influence the work reported in this paper.

4.	Duan G, Lan H, Shan W, Wu Y, Xu Q, Dong X, et al. Clinical effect of different doses of ciprofol for induction of general anesthesia in elderly patients: A randomized, controlled trial. Pharmacol Res Perspect. 2023;11(2):e01066. Epub 2023/02/23. doi: 10.1002/prp2.1066. PubMed PMID: 36811327; PubMed Central PMCID: PMCPMC9944862.

5.	Ding YY, Long YQ, Yang HT, Zhuang K, Ji FH, Peng K. Efficacy and safety of ciprofol for general anaesthesia induction in elderly patients undergoing major noncardiac surgery: A randomised controlled pilot trial. Eur J Anaesthesiol. 2022;39(12):960-3. doi: 10.1097/eja.0000000000001759. PubMed PMID: 36214498.

6.	Wang X, Wang X, Liu J, Zuo YX, Zhu QM, Wei XC, et al. Effects of ciprofol for the induction of general anesthesia in patients scheduled for elective surgery compared to propofol: a phase 3, multicenter, randomized, double-blind, comparative study. Eur Rev Med Pharmacol Sci. 2022;26(5):1607-17. Epub 2022/03/19. doi: 10.26355/eurrev_202203_28228. PubMed PMID: 35302207.

7.	Zhu Q, Luo Z, Wang X, Wang D, Li J, Wei X, et al. Efficacy and safety of ciprofol versus propofol for the induction of anesthesia in adult patients: a multicenter phase 2a clinical trial. Int J Clin Pharm. 2023;45(2):473-82. Epub 2023/01/22. doi: 10.1007/s11096-022-01529-x. PubMed PMID: 36680620; PubMed Central PMCID: PMCPMC10147789.

8.	Liang P, Dai M, Wang X, Wang D, Yang M, Lin X, et al. Efficacy and safety of HSK3486 vs. propofol for the induction and maintenance of general anaesthesia: A multicentre, single-blind, randomised, parallel-group, phase 3 clinical trial. Eur J Anaesthesiol. 2023. Epub 2023/01/18. doi: 10.1097/eja.0000000000001799. PubMed PMID: 36647565.

9.	Chen BZ, Yin XY, Jiang LH, Liu JH, Shi YY, Yuan BY. The efficacy and safety of ciprofol use for the induction of general anesthesia in patients undergoing gynecological surgery: a prospective randomized controlled study. BMC Anesthesiol. 2022;22(1):245. Epub 2022/08/04. doi: 10.1186/s12871-022-01782-7. PubMed PMID: 35922771; PubMed Central PMCID: PMCPMC9347095.

10.	Qin K, Qin WY, Ming SP, Ma XF, Du XK. Effect of ciprofol on induction and maintenance of general anesthesia in patients undergoing kidney transplantation. Eur Rev Med Pharmacol Sci. 2022;26(14):5063-71. Epub 2022/08/03. doi: 10.26355/eurrev_202207_29292. PubMed PMID: 35916802.

11.	Akhtar SMM, Fareed A, Ali M, Khan MS, Ali A, Mumtaz M, et al. Efficacy and safety of Ciprofol compared with Propofol during general anesthesia induction: A systematic review and meta-analysis of randomized controlled trials (RCT). J Clin Anesth. 2024;94:111425. Epub 2024/02/28. doi: 10.1016/j.jclinane.2024.111425. PubMed PMID: 38412619.

12.	Ozdogan HK, Cetinkunar S, Karateke F, Cetinalp S, Celik M, Ozyazici S. The effects of sevoflurane and desflurane on the hemodynamics and respiratory functions in laparoscopic sleeve gastrectomy. J Clin Anesth. 2016;35:441-5. Epub 2016/11/23. doi: 10.1016/j.jclinane.2016.08.028. PubMed PMID: 27871572.

***3. Comment:*** *The authors I think misunderstand the concept of uncertainty - this is another word for equipoise - in other words, are people genuinely uncertain about what to do in this situation - this needs to be evidenced in terms of current practice and why the results leading to approval cannot be extrapolated.*
**Response:** We totally understand the reviewer’s concern. Effects of ciprofol infusion on hemodynamics during induction and maintenance of anaesthesia and on postoperative recovery in patients undergoing thoracoscopic lobectomy are still unclear. According to previous studies, compared with propofol, ciprofol has less effect on circulation and respiration, and its application in general anesthesia sedation is not inferior to propofol. Now we are collecting data and haven't analysed the data yet, the results of the study is uncertain. As the reviewer mentioned,we can't conclude that ciprofol is a better choice of anesthetic agent compared with propofol.

We added the paragraph “It is not yet known whether ciprofol can prevent the stress response caused by surgery, provide sufficient sedation and maintain hemodynamic stability during thoracoscopic lobectomy.”

We deleted this paragraph “Conversely , because less effect on circulation and respiratory of ciprofol,quality of recovery in patients used ciprofol maybe is better than that of patients used propofol”. We revised as follow: “Moreover, because less effect on circulation and respiratory, fewer injection pain, ciprofol may be a promising alternative general anesthetics to propofol.” We have also added several more recent references. (page 19-20)

And here we did not list all the changes but marked in red in revised paper.

Thank you for your rigorous consideration once again.

***4.Comment:*** *For the sample size it needs to be specified that there will be 7 measurements per individual; what allowance for missing data is given in terms of not having all 7 in some cases? One of these is not an outcoem but a baseline measure as it is before induction so there are only 6 outcome timepoints here.*

**Response:** Thank you for your valuable comments. We totally understand the reviewer’s concern. Our data will be collected by independent researchers. The compliance of the participants is good. The data missing rate is low. If there is a missing value, we will use the method of multiple imputation for processing and conduct sensitivity analysis.

-------In section of **2.11 data management** ,we have added the following content “Data will be collected by independent researchers using Case Report Form(CRF). If there is a missing value, we will use the method of multiple imputation for processing and conduct sensitivity analysis.” (Line329-331,page 17)

We hope that the correction will meet with approval. Thank you once again.

***5.Comment:*** *The words shedding rate are still in the paper (line 1457 in the marked up copy).*
**Response:** We are very sorry for not writing clearly. We have changed the “shedding rate” to “drop out rate”.(Line 127, page7) We apologize for our errors. We thank the reviewer for pointing this out.

**6*.Comment:*** *Formatting of table 1 makes it look like the monitoring is in one arm only - please centralise the things to be done in both groups.*
**Response:** Thank you for your valuable comments. We have centralised the things to be done in both groups. (Page 12 ,Table 1)

**7*.Comment:*** *Section 2.7.1 seems to have redundancy about time points (there is also a &quot;the&quot; missing).*
**Response:** Thank you for your rigorous consideration. Referring to previous research [12], we delete the time points( such as : 60 minutes after the surgery begins,90 minutes after the surgery begins,120 minutes after the surgery begins).

Because our purpose is to study the hemodynamic changes during anesthesia induction and maintenance, in order to let the reader more clearly understand the setting of time points, we described the time points separately during induction and maintenance.

------**We revised as follows：** MAP and HR will be recorded during anesthesia induction，including the following time points: before induction (T_0_), after induction（T_1_ BIS≤60),immediately before intubation (T_2_),immediately after intubation(T_3_), 1,3, and 5 minutes after intubation(T_4_ ,T_5_,T_6_). (Line 235-241,page 12-13).

“MAP and HR will be recorded during anesthesia maintenance, including the following time points: when the patient is placed in the lateral decubitus position (OR_0_),when fiberoptic bronchoscope using for localization at the second time (OR_1_), immediately before the surgery (OR_2_), 1,3,5,and 30 minutes after surgical incision and at the end of surgery (OR_3_,OR_4_ ,OR_5_ ,OR_6_, OR_7_).”

(Line 242-246,page 13)

We hope that the correction will meet with approval. Thank you once again.

12.	Ozdogan HK, Cetinkunar S, Karateke F, Cetinalp S, Celik M, Ozyazici S. The effects of sevoflurane and desflurane on the hemodynamics and respiratory functions in laparoscopic sleeve gastrectomy. J Clin Anesth. 2016;35:441-5. Epub 2016/11/23. doi: 10.1016/j.jclinane.2016.08.028. PubMed PMID: 27871572.

**8*.Comment:*** *The literature gives a long range in terms of duration of the operation - how are operations lasting less than 2 hours to be dealt with in terms of time points?*
**Response:** Thank you for your valuable comments. We totally understand the reviewer’s concern. Referring to previous research [12], we carefully reconsidered the design of our study and deleted the time points such as “60 minutes after the surgery begins,90 minutes after the surgery begins,120 minutes after the surgery begins”. And we added an additional item in withdrawl criteria “the operation takes less than 30 minutes” , although in our research center, the surgery of thoracoscopic lobectomy lasts at least 30 minutes.**(line142-144,page7)**

----We revised as follow: “MAP and HR will be recorded during anesthesia maintenance, including the following time points: when the patient is placed in the lateral decubitus position (OR_0_),when fiberoptic bronchoscope using for localization at the second time (OR_1_), immediately before the surgery (OR_2_), 1,3,5,and 30 minutes after surgical incision and at the end of surgery (OR_3_,OR_4_ ,OR_5_ ,OR_6_, OR_7_).”

(Line 242-246,page 13)

12.	Ozdogan HK, Cetinkunar S, Karateke F, Cetinalp S, Celik M, Ozyazici S. The effects of sevoflurane and desflurane on the hemodynamics and respiratory functions in laparoscopic sleeve gastrectomy. J Clin Anesth. 2016;35:441-5. Epub 2016/11/23. doi: 10.1016/j.jclinane.2016.08.028. PubMed PMID: 27871572.

**9.*Comment:*** Thank you for clarifying 2.10.3 - presumably 2.10.4 really means that if one arm reaches 63 patients then the remaining patients will draw the other arm? As it stands it sounds like the trial will go on until there are equal numbers in each arm irrespective of the number required to get there.
**Response:** Thank you for your valuable comments. We apologize for our errors.

We have deleted this paragraph “If the number of cases in the two groups is not equal, the extra patients in one group can continue to receive random numbers until the two groups are equal.” ---We have revised as follow “If one arm reaches 63 patients ,the remaining patients will continue to receive random numbers until both groups reach at least 63 cases.”(line315-318,page 16)

**10.*Comment:*** Are the opaque envelopes to b prepared in advance or on the day? What safeguards are there to ensure that there is no foreknowledge?
 **Response:** We thank the reviewer for pointing this out. We are very sorry for not writing clearly. This is a single-blind study. To ensure the implementation of blind method , the opaque envelopes will be prepared in advance and handed over to the nurse who are responsible for preparing medications. The chief anesthesiologist of the patient and the nurse will open the sealed bag together on the day of surgery.

We revised as follows: “The generated random allocation will be placed in an opaque sealed bag in advance and handed over to the nurse who are responsible for preparing medications.The chief anesthesiologist of the patient and the nurse will open the sealed bag together on the day of surgery.”(Line 320-322,page 17)

----We appreciate for Reviewers’ warm work earnestly, and hope that the correction will meet with approval.

We have studied comments carefully and have made correction which we hope meet with approval.

We would like to thank the reviewers again for taking the time to review our manuscript.

Best Regards

Na Guo and Xiumei Song
