# Supplementary material for: Effects of ciprofol infusion on hemodynamics during induction and maintenance of anesthesia and on postoperative recovery in patients undergoing thoracoscopic lobectomy: Study protocol for a randomized, controlled trial
Source: PLoS One. 2024 Jul 10;19(7):e0305478. doi: 10.1371/journal.pone.0305478 (PMC11236111; doi:10.1371/journal.pone.0305478)
Supplement: Attachment — Submitted filename: Response to Reviewers..docx [file pone.0305478.s006.docx]

Dear Editor and Reviewers:

Thank you for your letter and for the reviewers’ comments concerning our manuscript entitled “Effects of ciprofol infusion on hemodynamics during induction and maintenance of anesthesia and on postoperative recovery in patients undergoing thoracoscopic lobectomy: study protocol for a randomized, controlled trial”.
(Manuscript ID: PONE-D-23-32710R2).

Those comments are all valuable and very helpful for revising and improving our manuscript, as well as the important guiding significance to our researches. We have studied comments carefully and have made correction which we hope meet with approval. The reviewers’ comments are laid out below in italicized font and specific concerns have been numbered. Our response is given in normal font.

Revised portion are marked in red in the paper.

***Journal Requirements:***

*Please review your reference list to ensure that it is complete and correct. If you have cited papers that have been retracted, please include the rationale for doing so in the manuscript text, or remove these references and replace them with relevant current references. Any changes to the reference list should be mentioned in the rebuttal letter that accompanies your revised manuscript. If you need to cite a retracted article, indicate the article’s retracted status in the References list and also include a citation and full reference for the retraction notice.*

**Response：**Thank you for your valuable comments. We have reviewed all the references to make sure that all the references are complete and correct. We also retrieved all the literatures in PubMed again. All the literatures that we cited have not been retracted. Thank you for your valuable suggestion again.

**Reviewer 1**

*Reviewer #1: Minor language polishing is still needed. There are several mistakes with punctuation, a few other minor mistakes like no spaces, irregular plural*

*- ciprofol may be a promising alternative general anesthetics to propofol[*

**Response to reviewer 1 :** We thank the reviewer for pointing this out. We are very sorry for our errors. We have carefully reviewed and revised the whole article. Such as add spaces, check the punctuation, singular, plural, and spelling errors.

We revised as follow

“also the effect on quality of recovery is still unknown” (page 18, Line361) ( spelling errors)

“ciprofol may be a promising alternative general anesthetic to propofol” (singular, plural errors) (page19,Line 373)

“We conduct the trial to investigate the effects of ciprofol on perioperative hemodynamics and on quality of recovery from anesthesia in patients undergoing thoracoscopic lobectomy.” (page19,Line 375). (spelling errors)

--And here we did not list the changes but marked in red in revised paper. Once again, thank you very much for your comments and suggestions.

**Reviewer 2**

*Reviewer #2: Thank you for your response.*

***Comment:*** *The sample size still does not say how many observations per patient the sample size is based upon - as such the sample size cannot be reconstructed. Please give all the details fed into PASS for repeated measures analysis.*

**Response:** Thank you for your valuable comments. We apologize for our errors. We seriously consulted the teachers of statistics at the center and rethought our sample size calculation process. We used a Repeated Measures analysis of variance (ANOVA) to calculate the sample size in the PASS 2021(v21.0.3). We observed 15 measurements of MAP ( T_0-6_ and OR_0-7_) per patient during induction and maintenance of anesthesia between the two groups.

We revised as follow:

“We used a Repeated Measures analysis of variance (ANOVA) to calculate the sample size in the PASS 2021(v21.0.3). According to the results of the preliminary experiment, We observed 15 measurements of MAP ( T_0-6_ and OR_0-7_) per patient during induction and maintenance of anesthesia between the two groups. The standard deviation of each group is approximately 13, and the autocorrelation coefficient between the adjacent measurement points of the same study participant is 0.5, the difference of the MAP between the two groups is 3. Type I error is 5% and the power is 80% in the pre-test. In the bilateral test, the sample size of the two groups is equal, and the sample size of each group is 54. Considering a 20% drop out rate, a sample size of 68 patients in each group is required. Thus, the final sample size is 136.”(Page 6-7, Line124-131)

We added the calculation process of the sample size in the supplementary materials.

We need to explain the reasons for the original calculation of sample size. We choose 7 measurements of MAP (T_0-6_) during induction of anesthesia to calculate the sample size at first, because our results of the preliminary experiment were consistent with those previously reported, and there were few studies about ciprofol for anesthesia maintenance.

We added the original calculation process of the sample size in the supplementary materials, too.

We choose 15 measurements during induction and maintenance of anesthesia and calculate the sample size again, the current method is more rigorous **(the final sample size is 136)**. Because the primary outcome that will be assessed in this study is the perioperative hemodynamic fluctuation (during induction and maintenance).

Fortunately, our trial is in the data collection stage, and we are grateful for the valuable suggestions and feedback from the reviewers, which are very helpful for us to improve our study. We hope the correction will meet with approval.

***Comment:*** *The opaque envelope approach is subject to subversion by opening envelopes out of order, or opening multiple envelopes and allocating out of order. There is an extensive literature on this and there needs to be evidence of how this is going to be policed - e.g. ensuring that envelopes are not opened until consent is given and that the consenting patient is actually treated according to allocation. At present one cannot guarantee robustness of the randomisation process.*

**Response:** We thank the reviewer for pointing this out. We delete this paragraph “The chief anesthesiologist of the patient and the nurse will open the sealed bag together on the day of surgery. ”

We revised as follow: “If the patient meets the inclusion and exclusion criteria, and has signed the informed consent, the anesthesiologist of the patient and the nurse will open the opaque sealed bag sequentially on the day of surgery and the consenting patient is treated according to allocation.” (page 15, Line 298-305)

----We appreciate for Reviewers’ warm work earnestly, and hope that the correction will meet with approval.

We have studied comments carefully and have made correction which we hope meet with approval.

We would like to thank the reviewers again for taking the time to review our manuscript.

Best Regards

Na Guo and Xiumei Song
